# Clinical Analysis: Aqueous-Deficient and Meibomian Gland Dysfunction in Patients With Primary Sjogren's Syndrome

**DOI:** 10.3389/fmed.2019.00291

**Published:** 2019-12-10

**Authors:** Yi Wang, Qiyu Qin, Bo Liu, Yana Fu, Lin Lin, Xiaodan Huang, Xiuming Jin

**Affiliations:** Eye Center, Second Affiliated Hospital, School of Medicine, Zhejiang University, Hangzhou, China

**Keywords:** dry eye, primary Sjogren's syndrome, meibomian gland dysfunction, lacrimal gland, aqueous-deficient

## Abstract

**Objective:** To examine the time course of aqueous-deficient and meibomian gland dysfunction (MGD) in patients with primary Sjogren's Syndrome (pSS).

**Methods:** This prospective study was conducted on pSS female patients in the Department of Rheumatism of the Second Affiliated Hospital, School of Medicine, Zhejiang University. The age-matched MGD female patients without pSS (non-SS-MGD) were recruited as MGD controls from the Eye Center of the Second Affiliated Hospital, School of Medicine, Zhejiang University. After providing written informed consent, the patients underwent an eye examination and completed an Ocular Surface Disease Index questionnaire that assessed the symptoms of dry eye disease. The subjects were evaluated using Schirmer I test (SIt), tear meniscus height (TMH), noninvasive keratographic break-up time (NIKBUT), corneal fluorescein staining (CFS), and meibomian gland evaluation (meibomian gland infrared, lid margin score, expressible meibomian glands number and the secretions quality). The patients were divided into two groups: early stage (≤3 years) and late stage (>3 years) according to their medical history of dry eye. The data were analyzed using SPSS 20.0.

**Results:** There were 49 pSS and 52 non-SS-MGD female patients enrolled in this study from 1 January 2018 to 30 December 2018. There were no differences in age (49.38 ± 10.32 and 48.69 ± 13.57 years) and dry eye medical history (48.44 ± 40.16 and 47.79 ± 37.85 months) between the two groups. When the medical history was ≤3 years, the average SIt and TMH of the pSS patients were significantly smaller than those of the patients with MGD. However, the signs related to the MGD did not show a significant difference between the two groups. When the medical history was >3 years, both the SIt and TMH and the signs related to MGD in pSS group were significantly more severe than the MGD group.

**Conclusions:** Our results demonstrated that 3 years may be an important time node for the dry eye development in pSS patients, before this, the lacrimal glands received a greater influence, and then the meibomian glands began to be greatly affected.

## Introduction

Primary Sjogren's Syndrome (pSS) is a complex and currently incurable chronic inflammatory systemic autoimmune disease characterized by invasion of exocrine glands. It is one of the leading causes of aqueous-deficient dry eye disease (DED) in the world (1). As the disease develops, it causes multiple organ damage in the kidneys, lacrimal glands, salivary glands, and may result in cutaneous vasculitis demonstrated by cutaneous purpura and/or rash. The main clinical manifestations of pSS are dry eye, dry mouth, mild fever, weakness, and other systemic symptoms (2). It is generally considered that the pathogenesis of DED in patients with primary Sjogren's Syndrome is the abnormal tears secretion caused by lacrimal gland damage, which leads to eye discomfort and visual dysfunction (3).

The infiltration of lymphocytes in exocrine glands (i.e., salivary and lacrimal glands) is a typical pathological feature of pSS. Inflammatory cell invasion and the secretion of inflammatory factors destroy the lacrimal gland cells and secretory ducts, and may damage the tarsal gland, resulting in abnormal tear quality and quantity, which cause dry eyes (4). Traditionally, the mechanisms operative in dry eye are attributed to either impaired lacrimal production (aqueous-deficient dry eye) or excessive evaporation as a result of meibomian gland dysfunction (MGD), the most common form of evaporative dry eye (5–8). In clinical experience, a mixed type of dry eye has been identified in pSS patients (9). Previous study demonstrated that pSS and sSS (secondary Sjogren's Syndrome) patients have both aqueous-deficient and evaporative DED. They showed that the effects of pSS on the ocular surface are not limited to lacrimal glands but are also associated with the meibomian glands (3, 10). However, the chronological order in which the lacrimal glands and the meibomian glands are affected in the patients with pSS is still unknown.

## Methods

This study was approved by the Ethics Committee at the Affiliated Second Hospital, School of Medicine, Zhejiang University in Hangzhou, China. All the procedures adhered to the tenets of the Declaration of Helsinki. A written informed consent was obtained from each participant.

### Patients

Female patients with pSS were recruited in the Department of Rheumatism of the Second Affiliated Hospital, School of Medicine, Zhejiang University. Meibomian gland dysfunction (MGD) without SS (non-SS-MGD) age-matched female patients were recruited as MGD controls from the Eye Center at the Second Affiliated Hospital, School of Medicine, Zhejiang University.

The inclusion criteria for the pSS subjects were as follows: (a) age 20–80 years, female; (b) conformed to the diagnostic criteria of primary Sjogren's Syndrome. The diagnostic criteria for primary Sjogren's Syndrome are based on the 2016 American College of Rheumatology/European League Against Rheumatism classification criteria for primary Sjogren's Syndrome (11).

The inclusion criteria for the non-SS-MGD subjects were as follows: (a) age 20–80 years, female; (b) conformed to the diagnostic criteria of MGD. The diagnostic criteria for MGD are based on physical signs, symptoms, and corresponding auxiliary examination results for comprehensive evaluation.

1. Abnormal opening of the scar margin and meibomian glands;
2. Abnormal secretion of oxime ester;
3. With ocular symptoms;
4. Loss of meibomian glands;
5. The thickness of the lipid layer is abnormal.

Patients with either item 1 or 2 can be diagnosed as having the meibomian gland abnormalities, and combined with the third eye symptom, the symptomatic person can be diagnosed as MGD. Item 4 and 5 are strengthening diagnostic indicators (12).

The exclusion criteria for the pSS and non-SS-MGD subjects were as follows: (a) ophthalmic surgery within 3 months; (b) wearing contact lenses; (c) patients with dry eye caused by antibiotic eye drops; (d) eyes subjected to chemical or heat burn; (e) severe systemic diseases, such as heart and brain diseases and mental disorders; (f) refusal to sign the informed consent form required to participate in this study.

After the patients signed the informed consent form, the general data on the subjects were collected: gender, age, and medical history. The Ocular Surface Disease Index questionnaire (OSDI scale), tear meniscus height (TMH), non-invasive keratographic break-up time (NIKBUT), Schirmer test (Schirmer I test, SIt), meibomian gland evaluation (MGE), and the corneal fluorescein staining (CFS) were also evaluated.

### Clinical and Symptoms Evaluation

#### OSDI

The international OSDI integral method was used to evaluate the patients' responses to the eye symptom (13). A total of 12 symptoms were evaluated: photophobia, foreign body sensation, eye pain, blurred vision, low vision, inability to read, inability to drive at night, inability to work on a computer, inability to watch TV, eye discomfort during wind, eye discomfort when the environment is dry and eye discomfort when air conditioning is turned on. The symptoms rated on the following scale: never = 0, occasionally = 1, non-continuous = 2, continuous = 3, and always = 4 points. All 12 questions did not have to be answered (The scenarios described in the questionnaire are not suitable for all groups of people, for instance, people who do not watch television are not required to answer the question about whether their eye discomfort influenced their viewing). The formula for calculating OSDI points is: the total number of points above × 25 / the number of questions answered by the patient, and the total score is 100 points.

#### Tear Meniscus Height

The lower TMH was examined using the ocular surface analyzer (OCULUS). This examination was repeated three times for each eye, and the average TMH was recorded. The normal height of the lower TMH is 0.2~0.3 mm (14).

#### NIKBUT Measurement

NIKBUT is an objective index to evaluate the stability of tear film. The patients were asked to blink their eyes three times and then to keep their eyes open as long as possible. The time from the last complete blink to the first perturbation of rings projected onto the surface of the cornea was measured, which represented the NIKBUT. Both eyes were tested in each patient. This procedure was repeated three times for each eye, and the average time was recorded. An NIKBUT value of <5 s was accepted as “abnormal,” and an NIKBUT ≥10 s was standard. An NIKBUT of <10 s indicates an unstable tear film and a lack of mucin in the tear fluid, which may result from severe damage or loss of goblet cells in the conjunctiva (15).

#### Schirmer I Test

Under the condition of no surface anesthesia, a 35 × 5 mm filter strip was placed in the conjunctival sac under the eyes, and the wetting length was recorded after 5 min. Wetting values ≥10 mm/5 min is normal (14).

#### Meibomian Glands Evaluation (MGE)

The infrared imaging of the meibomian glands (16) was conducted as follows: the palpebral glands of the upper and lower eyelids were photographed using the eye table comprehensive analyzer, and the areas of the palpebral glands were outlined using relevant software (OCULUS). Finally, the missing areas of the upper and lower eyelid meibomian glands were calculated by the software automatically.

The lid margin score was obtained as follows: we evaluated the abnormality of the meibomian gland orifices, lid tenderness, and telangiectasia on a scale from 0 to 4, where 0 = absent and 4 = the most severe (17).

The number of expressible meibomian glands was assessed as follows: meibomian gland expressibility was assessed using the Meibomian Gland Evaluator. Each of five meibomian glands in the nasal, middle, and bitamporal parts of the upper eyelid was assessed on a scale from 0 to 3: 0 = all 5 glands expressible; 1 = 3–4 glands expressible; 2 = 1–2 glands expressible; 3 = no glands expressible. The total scores ranged from 0 to 9.

The quality of the meibomian gland secretions was assessed by eye examiners as follows: The lower and upper eyelids were divided into three parts: nasal, bitamporal, and middle. Each part has 5 glands. The scores for the meibum characteristics secreted by each gland were as follows: 0 = no secretion drainage; 1 = inspissated toothpaste-like lipid; 2 = viscous opaque or yellow lipid; 3 = liquid clear lipid. The scores for each expressed orifice in three parts of the eyelids were summed to obtain the final score (0–90) for the right and left eyes (17).

#### Corneal Fluorescein Staining

The procedure for scoring corneal fluorescein staining (CFS) was based on previous reports (18). The cornea was equally divided into the upper, lower, nasal, bitamporal and middle, and five sections. The score for each section was recorded after staining as follows: 0 = no punctate staining; 1 = less than half staining; 2 = more than half staining; 3 = whole staining; and cumulative score for each eye ranged from 0 to 15. Fluorescein staining showed corneal or conjunctival epithelial cells injury or ulcer. The normal corneal epithelium is smooth and intact, so it cannot be stained by fluorescein (19).

### Statistical Treatment

SPSS22.0 statistics software was used to analyze the data. The experimental data were represented by means ± SD. The correlation between the indexes was statistically analyzed using single factor analysis of variance. *P* < 0.05 was considered as statistically significant.

## Results

There were 49 pSS female patients and 52 non-SS-MGD age-matched female patients enrolled in this study from 1 January 2018 to 30 December 2018. The average ages of the pSS patients and the non-SS-MGD patients were 49.38 ± 10.32 and 48.69 ± 13.57 years, respectively. There were no differences in age and dry eye medical history (48.44 ± 40.16 and 47.79 ± 37.85 months) between the two groups (Table 1). The antibody status of 49 pSS patients indicated that the highest positive rate was antinuclear antibody (91.8%), followed by anti-Ro-52 antibody (85.7%), anti-SSA antibody (81.6%), anti-SS-B antibody (26.5%), and anti-dsDNA antibody (4.1%). 31 (63.3%) pSS patients received biopsy of salivary glands, and the unstimulated whole salivary flow was measured in 25 patients (51.02%), with only 2 patients did parotid sialography.

**Table 1:** The characteristics of patients with pSS and non-SS-MGD.

**Variables**		**pSS**	**Non-SS-MGD**
Female		49	52
Age (years)		49.38 ± 10.32	48.69 ± 13.57
Medical history (months)		48.44 ± 40.16	47.79 ± 37.85
OSDI		29.76 ± 21.05	19.77 ± 9.93^b^
Schirmer I test (mm)	OD	4.19 ± 3.95	7.58 ± 4.91^b^
	OS	4.21 ± 3.87	7.61 ± 5.04^b^
BUT (s)	OD	3.26 ± 2.71	6.77 ± 3.86^b^
	OS	3.24 ± 2.75	6.69 ± 3.74^b^
CFS (score)	OD	8.25 ± 5.71	4.85 ± 3.36 ^b^
	OS	8.17 ± 4.87	4.87 ± 3.42^b^
TMH (mm)	OD	0.08 ± 0.12	0.13 ± 0.18^a^
	OS	0.08 ± 0.11	0.12 ± 0.17^a^
Meibomian gland atrophy areas	OD	32.09 ± 12.33	20.55 ± 7.05^b^
	OS	33.43 ± 13.02	21.38 ± 7.31^b^
Lid margin score	OD	2.84 ± 0.72	1.26 ± 0.39^a^
	OS	2.62 ± 0.68	1.21 ± 0.41^a^
Expressible meibomian glands number	OD	3.16 ± 0.71	5.21 ± 0.81 ^a^
	OS	3.22 ± 0.69	5.26 ± 0.79 ^a^
Meibomian gland secretions quality	OD	3.35 ± 0.57	5.54 ± 1.08^a^
	OS	3.33 ± 0.53	5.61 ± 1.11^a^

*Symptoms and DED related examinations in the patients with pSS compared to non-SS-related MGD. Values equal the mean ± SE. DED, dry eye disease; MGD, meibomian gland dysfunction; pSS, primary Sjögren syndrome; OSDI, Ocular Surface Disease Index; CFS, corneal fluorescein staining*.

a, b *Significantly (^a^p < 0.05, ^b^p ≤ 0.01) greater than in the controls*.

The OSDI scores of pSS patients were significantly higher than non-SS-MGD (29.76 ± 21.05 vs. 19.77 ± 9.93, *p* ≤ 0.01; Table 1), which showed that pSS was associated with a significant increase in the severity and frequency of ocular symptoms.

### Impact of pSS on the Anterior Segment

Compared with non-SS-MGD patients, pSS patients had significantly decreased tear secretion (SIt: OD 4.19 ± 3.95 vs. 7.58 ± 4.91 mm, OS 4.21 ± 3.87 vs. 7.61 ± 5.04 mm, all *p* ≤ 0.01; TMH: OD 0.08 ± 0.12 vs. 0.13 ± 0.18 mm, OS 0.08 ± 0.11 vs. 0.12 ± 0.17 mm, all *p* < 0.05; Table 1) and NIKBUT (OD 3.26 ± 2.71 vs. 6.77 ± 3.86 s, OS 3.24 ± 2.75 vs. 6.69 ± 3.74 s, all *p* ≤ 0.01; Table 1). In addition, compared to the non-SS-MGD patients, CFS (OD 8.25 ± 5.71 vs. 4. 85 ± 3.36, OS 8.17 ± 4.87 vs. 4.87 ± 3.42, all *p* ≤ 0.01; Table 1) was significantly higher in the pSS patients. Overall, the extent of the aqueous tear deficiency and ocular surface damage was far greater in the pSS patients than in the non-SS-MGD patients.

The evaluation of the meibomian glands showed that the lid margin score and the meibomian gland atrophy areas were significantly increased (Lid margin score: OD 2.84 ± 0.72 vs. 1.26 ± 0.39, OS 2.62 ± 0.68 vs. 1.21 ± 0.41, all *p* < 0.05; Meibomian gland atrophy areas: OD 32.09 ± 12.33 vs. 20.55 ± 7.05, OS 33.43 ± 13.02 vs. 21.38 ± 7.31, all *p* ≤ 0.01; Table 1) and that the number of expressible meibomian glands and the quality of the meibomian gland secretions were significantly reduced in the pSS patients compared to the non-SS patients with MGD (Expressible meibomian glands number: OD 3.16 ± 0.71 vs. 5.21 ± 0.81, OS 3.22 ± 0.69 vs. 6.26 ± 0.79, all *p* < 0.05; Meibomian gland secretions quality: OD 3.35 ± 0.57 vs. 5.54 ± 1.08, OS 3.33 ± 0.57 vs. 5.61 ± 1.11, all *p* < 0.05; Table 1).

### The Different Impact of pSS on the Anterior Segment According to the Medical History

When the medical history was ≤3 years, the average SIt and TMH of the pSS patients were significantly shorter than that of non-SS-MGD patients (SIt: OD 4.78 ± 0.79 vs. 7.21 ± 0.91 mm, OS 4.80 ± 0.81 vs. 7.28 ± 0.89 mm, all *p* ≤ 0.01; TMH: OD 0.09 ± 0.10 vs. 0.13 ± 0.16 mm, OS 0.09 ± 0.11 vs. 0.12 ± 0.18 mm, all *p* < 0.05; Table 2). However, the signs related to MGD did not show a significant difference between the pSS and the non-SS-MGD patients (Table 3).

**Table 2:** The characteristics of patients with pSS and non-SS-MGD according medical history.

**Variables**		**pSS (≤3y)**	**Non-SS-MGD (≤3y)**	**pSS (>3y)**	**Non-SS-MGD (>3y)**
Female		26	27	23	25
Age (years)		47.26 ± 11.18	46.73 ± 11.39	51.55 ± 8.86	49.91 ± 12.35
Medical history (months)		17.91 ± 14.21	16.97 ± 15.36	82.96 ± 30.68	80.66 ± 31.72
OSDI		25.56 ± 10.01	19.46 ± 8.44^b^	31.73 ± 9.85	20.49 ± 7.71^b^
Schirmer I test (mm)	OD	4.78 ± 0.79	7.21 ± 0.91^b^	3.11 ± 0.75	7.91 ± 0.93^b^
	OS	4.80 ± 0.81	7.28 ± 0.89^b^	3.18 ± 0.69	7.98 ± 0.91^b^
BUT (s)	OD	4.21 ± 2.17	6.97 ± 3.79^b^	2.31 ± 1.85	6.38 ± 3.81^b^
	OS	4.19 ± 2.09	7.01 ± 3.79^b^	2.23 ± 1.72	6.09 ± 3.54^b^
CFS (score)	OD	7.12 ± 5.33	4.26 ± 2.15^b^	9.31 ± 5.11	5.12 ± 2.98^b^
	OS	7.09 ± 5.26	4.31 ± 3.32^b^	9.27 ± 5.37	5.11 ± 2.95^b^
TMH (mm)	OD	0.09 ± 0.10	0.13 ± 0.16^a^	0.07 ± 0.12	0.13 ± 0.19^a^
	OS	0.09 ± 0.11	0.12 ± 0.18^a^	0.07 ± 0.11	0.12 ± 0.16^a^

*Symptoms and DED related examinations in the patients with pSS compared to non-SS-related MGD. Values equal the mean ± SE. DED, dry eye disease; MGD, meibomian gland dysfunction; pSS, primary Sjögren syndrome; OSDI, Ocular Surface Disease Index*.

a, b *Significantly (^a^p < 0.05, ^b^p ≤ 0.01) greater than in the controls*.

**Table 3:** The MGE characteristics of patients with pSS and non-SS-MGD according medical history.

**Variables**		**pSS (≤3y)**	**Non-SS-MGD (≤3y)**	**pSS (>3y)**	**Non-SS-MGD (>3y)**
Female		26	27	23	25
Meibomian gland atrophy areas	OD	21.22 ± 12.33	20.15 ± 7.12	40.29 ± 12.33	21.65 ± 7.21^b^
	OS	21.18 ± 13.02	20.21 ± 7.27	40.53 ± 13.02	22.76 ± 7.41^b^
Lid margin score	OD	1.24 ± 0.72	1.18 ± 0.19	4.43 ± 0.72	1.34 ± 0.39^a^
	OS	1.22 ± 0.68	1.11 ± 0.41	4.06 ± 0.68	1.33 ± 0.41^a^
Expressible meibomian glands number	OD	4.09 ± 0.77	5.09 ± 0.87	2.25 ± 0.62	5.51 ± 0.90^a^
	OS	4.02 ± 0.71	5.11 ± 0.87	2.28 ± 0.59	5.41 ± 0.89^a^
Meibomian gland secretions quality	OD	4.61 ± 0.57	5.76 ± 1.12	2.11 ± 0.57	5.26 ± 0.98^a^
	OS	4.55 ± 0.53	5.81 ± 1.13	2.13 ± 0.53	5.37 ± 1.01^a^

*Meibomian gland related examination in the patients with pSS compared to non-SS-related MGD. Values equal the mean ± SE. DED, dry eye disease; MGD, meibomian gland dysfunction; pSS, primary Sjögren syndrome; OSDI, Ocular Surface Disease Index*.

a, b *Significantly (^a^p < 0.05, ^b^p ≤ 0.01) greater than in the controls*.

When the medical history was >3 years, both the SIt and TMH (SIt: OD 3.11 ± 0.75 vs. 7.91 ± 0.93 mm, OS 3.18 ± 0.69 vs. 7.98 ± 0.91 mm, all *p* ≤ 0.01; TMH: OD 0.07 ± 0.12 vs. 0.13 ± 0.19 mm, OS 0.07 ± 0.11 vs. 0.12 ± 0.16 mm, all *p* < 0.05; Table 2) and the signs related to MGD in the pSS patients were significantly more severe than in the non-SS-MGD patients (Lid margin score: OD 4.43 ± 0.72 vs. 1.34 ± 0.39, OS 4.06 ± 0.68 vs. 1.33 ± 0.41, all *p* < 0.05; Meibomian gland atrophy areas: OD 40.29 ± 12.33 vs. 21.65 ± 7.21, OS 40.53 ± 13.02 vs. 22.76 ± 7.41, all *p* ≤ 0.01; Expressible meibomian glands number: OD 2.25 ± 0.62 vs. 05.51 ± 0.90, OS 2.28 ± 0.59 vs. 5.41 ± 0.89, all *p* < 0.05; Meibomian gland secretions quality: OD 2.11 ± 0.57 vs. 5.26 ± 0.98, OS 2.13 ± 0.53 vs. 5.37 ± 1.01, all *p* < 0.05; Table 3).

## Discussion

The results we obtained in this study indicated that 3 years may be an important time node for the dry eye development in the pSS patients. Before this, the lacrimal gland received a greater influence, and then the meibomian glands began to be greatly affected. The patients with pSS, but not those with non-SS MGD, had more significant problems with poor and blurred vision.

Studies have reported that the volume (20), stability, lipid layer (21) and surface activity (22) of tears in SS patients decreased, while tear film evaporation (23), ocular surface damage (22) and symptoms of DED (8) increased. Researches also revealed that the meibomian glands have decreased expression and quality (24), and meibomian gland dropout and dysfunction are increased in subjects with Sjogren's Syndrome (10, 21). However, up to now, no study has unveiled the relationship between the two important glands that were closely associated with dry eye, lacrimal and meibomian glands, in pSS patients. Thus, our study was conducted to investigate the temporal relation of the involvement between lacrimal and meibomian glands in pSS patients and may demonstrate the characteristics of exocrine glands damage in the pathogenesis of pSS.

Here, we found that compared to dry eye patients with only MGD, the pSS dry eye patients suffered from even more serious signs and symptoms, which may significantly threaten the pSS patients' life quality as well as their personal and economic well-being. It was found that the neural innervation lost may occur in areas of extensive inflammation, which was observed in the salivary glands of the pSS patients (25). The lymphocyte infiltration in the conjunctiva and exocrine gland, along with the decreased neural innervation may promote ocular surface damage, contributing to even more severe symptoms and signs in pSS patients. Similar with other researches, we found that apart from the lacrimal glands damage, pSS patients also had meibomian gland dysfunction. Several mechanisms may contribute to the pSS related MGD in women, such as androgen deficiency targeted by the immune system in pSS, cytokine-induced disruption of neural-meibomian gland epithelial cell interactions, and secondary to the aqueous tear deficiency (4). Besides, we here hypothesized that some symptoms of pSS patients, such as disturbed sleep, depressed mood, and fatigue may also lead to MGD because of the absence of blinking, as all the mentioned symptoms could result in less secretion of dopamine, which was an essential chemical substance that was positively correlated with the rate of blinking (6, 26). Further research is required to elucidate the underlying mechanisms that promote these pathological conditions of MGD in pSS patients.

More importantly, we found in patients with a medical history of dry eye less than or equal to 3 years, the average SIt and TMH, two indexes that mainly reflect the volume of tears among the ocular surface and intimately represent the condition of lacrimal glands of pSS patients, were significantly shorter than that of the patients with MGD. However, no differences of the signs related to the MGD were found between the two groups. When the medical history was more than 3 years, all evaluated indexes related to tear volume or meibomian gland dysfunction were significantly more severe in pSS group than non-SS-MGD group. Unlike the lacrimal glands, which were badly damaged from the start, the meibomian glands in pSS patients will receive a greater impairment when the medical history of dry eye was more than 3 years. The time gap of the enrollment of lacrimal glands and meibomian glands in pSS patients partly proved the possibility that the dysfunction of meibomian glands may occur secondary to lacrimal glands damage caused aqueous tear deficiency. But the specific pathogenic mechanisms for the different effects on meibomian glands and lacrimal glands in pSS patients awaiting further in-depth research.

Despite positive outcomes, there were still certain limitations of the present research: First, no normal control subjects without dry eye were enrolled in this study because of the time, energy and financial constraints. There were a few healthy volunteers who have no dry eyes in our normal clinical practice, and it was difficult to obtain a control group that was sufficiently matched with age. But we provided the normal range of all evaluated indexes, hoping to help readers better understand the extent of indexes abnormalities in pSS patients and non-SS-MGD patients. Second, the participants in the study were comparatively small, a more detailed research that recruits a larger number of cases is still needed. Third, 2 patients (4.10%) with positive anti-dsDNA antibody were enrolled in our cohort. Anti-dsDNA antibody is a typical antibody for systemic lupus erythematosus, but we really did not find any definite secondary diseases or related symptoms among these two patients. Still, we cannot exclude the possibility that patients with sSS may enroll in our study. But as this part of patients only took up a small percentage of our pSS patients, we think they did not have the potential to shake up our final results.

In summary, the results of the present study suggested that pSS patients have both aqueous-deficient and evaporative DED. When the medical history of dry eye was less than or equal to 3 years, the lacrimal gland received a greater influence, and then the meibomian glands began to be greatly affected in the pSS patients. Thus, the lacrimal glands may be destroyed earlier than the meibomian glands in the pSS patients.

## Data Availability Statement

The raw data supporting the conclusions of this manuscript will be made available by the authors, without undue reservation, to any qualified researcher.

## Author Contributions

All authors listed have made a substantial, direct and intellectual contribution to the work, and approved it for publication.

### Conflict of Interest

The authors declare that the research was conducted in the absence of any commercial or financial relationships that could be construed as a potential conflict of interest.
